# Hospital and Institutional News

**Published:** 1916-04-29

**Authors:** 


### HOSPITAL AND INSTITUTIONAL NEWS.

### OUR SPECIAL NUMBER.

Next week's issue of The Hospital will be the annual May Special Number. Special Numbers in these days of paper scarcity, labour shortage, and staff difficulties, are not without their problems; but the issue of The Hospital of May 6 will, we hope, equal in interest, if it does not even excel, that of previous Special Numbers. Two new series of special importance will be commenced in this issue, the first on "Temperament in the Individual," and the second on "The Institutional Treatment of Consumption." It will contain some hospital plans of real interest, including those of a great Canadian general hospital and a special hospital in Calcutta. Some notable books will be dealt with in the Monthly Review Supplement appearing with this number.

### HONOURS FOR THE WITTENBERG DOCTORS.

With commendable promptitude the three R.A.M.C. officers who survived the horrors of Wittenberg Camp have been gazetted to decorations of honour. Major H. E. Priestley receives the C.M.G.; Captain A. C. Vidal and temporary Captain J. L. Lauder the D.S.O. Can these awards be considered adequate? We consider not. According to rumour, the constitution of one at least of these gallant doctors has been completely ruined by his experiences; and the wonder is that anyone came through that appalling epidemic alive. In view of what has been published about the no less conspicuous gallantry of the rank and file at Wittenberg, it would be gratifying to see a list of awards of the Medal for Distinguished Conduct to those who were most conspicuous for courage and endurance in helping the British medical officers to combat the epidemic. The feeble denial issued by the German Government in response to the disclosures made in this country makes the matter, if possible, worse: the chief rebuttal is the statement that an American inspector's report does not bear out the statements of our officers. Naturally it does not, for Americans were kept strictly away until the epidemic was over.

### TYPHUS IN OTHER GERMAN PRISONS.

Officially, the British Government has "no reason to suppose" that the horrors of Wittenberg have been paralleled elsewhere in Germany: a comforting assurance if officialdom could be relied upon, but one only too probably quite fallacious, if certain information published in the *Field* is accurate. That newspaper gives the names of five separate camps in Germany where extensive epidemics of typhus are known to have occurred. At some there are no British prisoners, and at others a few; which may explain why less has been heard of them than at Wittenberg. At all of them, however, the epidemics seem to have been deliberately fostered by the German authorities; and the same purposive policy of withdrawing all German doctors and orderlies, and sending (wrongfully) imprisoned captive doctors to the camps was followed. Not only so, the lack of all facilities for proper treatment, of good and sufficient food, of protection against climate, and the other horrible features of the Wittenberg crime, seem all to have been duplicated in these five camps. One French doctor served in turn in three of these camps, and the descriptions he gives of the sufferings of the patients, totally neglected as they were by their gaolers, are comparable with what we know of the scenes witnessed by Major Priestley and his comrades. The mortality rate of these typhus epidemics seems, nevertheless, to have been fairly low, at least at Gardelegen, where two R.A.M.C. officers took part in the work of combating the disease.

### ST. GEORGE'S HOSPITAL SECRETARYSHIP.

Mr. Henry Wingrove is shortly retiring after a service of thirty years at St. George's Hospital, and will be succeeded, we understand, by Mr. James M. Churchfield the assistant secretary. Mr. Wingrove served for many years under Mr. C. L. Todd, a great secretary in his time. A resident medical officer two years later was entrusted with rather full administrative functions, and Mr. Wingrove was then appointed secretary to the House Committee, his title in recent years being Secretary.

### INVALID WAR PRISONERS IN SWITZERLAND.

The humane and beneficent arrangement long ago concluded between the French, German, and Swiss Governments, whereby invalid prisoners of war of the two former nationalities may be interned under due safeguards in healthy sanatoria in Switzerland, has some prospects of being extended shortly to British prisoners in Germany and reciprocally to German prisoners in Britain. It has been broadly hinted by one well-known M.P. that the long delay over the fruition of this splendid scheme is attributable to unreasonable stipulations by some Department or other of our own Government, a charge so serious that we hesitate to believe it. From what we know of the wanton cruelty and deliberate mal-hygiene of German prison camps, it is all too probable that this measure of relief will be too late for hundreds of our gallant fellows: indeed, we know only too well that the discrimination practised against British prisoners has been most deadly in spreading tuberculosis and similar prison diseases. There is the more necessity for a speedy issue of the negotiations, and for short work to be made of any obstructionist on this side of the Channel, if such a callous and unpatriotic person really exists.

### THE SCHEDULE OF DISEASES.

Those diseases which may afford to the sufferer the benefits of internment in Switzerland under the Franco-German arrangement form a fairly comprehensive list. The selection, be it said, is done by Swiss doctors; and a comprehensive hotch-potch clause gives them a pretty wide discretion, for it is provided that they may select "isolated cases comprised in none of the categories, but necessitating, in the opinion of the Commission, immediate internment in Switzerland." Tuberculosis in any stage whatever, and syphilis in advanced stages, are admissible: but all other contagious maladies, during their period of transmissibility, are expressly excluded, as are also chronic alcoholism and mental derangement. We should imagine that there are more cases of the last than of the next last disorder in the German prison camps. Many of the categories are such as would, in the ordinary way, result in exchange under the totally disabled system; and there are one or two peculiar items which are a little difficult to understand. Thus loss of a limb entitles officers and non-commissioned officers to be sent to Switzerland: can it be that privates in this predicament are sent home, but that officers and N.C.O.s are not? Perhaps when the scheme for our own men gets settled, such anomalous provisions will be found to be amended.

### THE CHEERFUL WOUNDED IN FRANCE.

According to Major Dr. W. A. Chapple, M.P., who is serving as a surgeon at the Front, the hospital wards are often the jolliest places out there; and the same has been remarked by many other observers with similar opportunities of judging. In a letter home Dr. Chapple remarks that there is less actual suffering by the wounded than would be imagined, many wounds producing no pain whatever. A fine type of a "Kiltie" showed the doctor a half-crown that he had worn in a belt round his waist. One-third of it was punched right out by a shrapnel bullet with which he was hit, yet he felt no pain whatever, and only knew of his wound when he saw blood on his bare knee. People often, says Dr. Chapple, have an exaggerated notion of the mental sufferings of wounded men. Their cheerfulness is amazing, and a hospital ward is often the jolliest place one can enter. "The very finest traits in human character are to be found in the hospital wards," he adds. "'Tommy' is not only tender and sympathetic; he is instinctively chivalrous. It is often proud comment amongst us surgeons how immune our nurses are from offence."

### OUR OVERSEA SOLDIERS.

Experience testifies, as one Commandant of a large hospital at Malta puts it, that the Australians are sometimes troublesome, but are invariably most helpful when there is work to be done. The truth appears to be that the open and free life of the Colonies has accustomed the men to wander at will over great tracts of country and to be free and unrestrained. To put such men under narrow and strict discipline, hampered by innumerable rules, is to court difficulties. Where, however, our soldiers from beyond the seas are treated with perfect frankness, put upon their honour, and asked to co-operate with the officers in securing discipline in the interest of everybody, there it is found that everything works with a minimum of difficulty and trouble. Woodcote Park is well worth a visit. The arrangements for its working, and for the organisation of sports and games and relaxation, under Lord Killanin, are really admirable.

### A UNANIMOUS VERDICT.

Matrons and sisters testify to the admirable patients which our Colonial warriors prove themselves to be when inmates of a voluntary hospital where the whole of the arrangements have been carefully thought out, and every effort is made to provide cheerful surroundings, ample comfort, and the quickest possible recovery. The effect on the men, both British and Colonial, of association together in camp during their time of convalescence or when under hospital care has been, on the whole, admirable. The experience gained is already sufficient to prove that in the future this system should tend to promote the best interests of the nation and the Empire.

### BRING THE MEN TOGETHER.

Attention has been drawn in the *Times* to the importance which is properly attached to every opportunity being taken to utilise the time during which wounded and sick soldiers from all parts of the Empire are under treatment in the Motherland to bring them together and mix them up as much as possible, so that they may get to know and appreciate each other's points of view, to understand the working of each other's minds, become good and intimate comrades, and in this way to consolidate the Empire through its warriors. The idea is a good one, and where it has been attempted with understanding, the results have been admirable. At the camp at Woodcote Park, Epsom, which has accommodation for 4,000 men, there are amongst the patients, in addition to British soldiers, large numbers from Australia, New Zealand, Canada, and South Africa. The officers' mess at this camp is full of interest, for the officers have been drawn from all parts of the Empire, with a result which has proved so beneficial and helpful to every member of it that it has won approval from all for the system which has brought it about.

### A CANADIAN HOSPITAL FOR FRENCH WOUNDED.

An interesting description reaches this country of the Canadian Hospital at St. Cloud, which will shortly rank as the second in France in size. Situated in the delightful grounds of the St. Cloud racecourse, the hospital is said to be one of the brightest and best across the Channel. At present the building can accommodate about 400 patients, but extensions are being made, and one of the latest additions is what is termed a "shed" near the grand-stand which contains twenty cots. Before long the hospital will be further extended so as to receive 1,400 patients. Beside the matron in charge there is a medical staff of twenty doctors and a nursing staff of thirty-three. When the hospital becomes the second largest in France, it is stated, there will be an addition to the staff of fifty medical men and seventy-two nurses: if this statement is true the hospital is being grossly overstaffed in medical officers. It is intended then to place all the wounded under canvas, as experience has shown that this is better for the men than wooden huts. Floored tents are being erected in the racecourse grounds, the inside canvas being a drab yellow colour, a shade which is found more restful for the eyes than green. The dining, smoking, and club rooms behind the grand-stand have been transformed into recreation, writing, and sitting rooms. Nearly all the wounded at present in the hospital are from the Verdun Front. As most of the surgeons and nurses speak French fluently, and French cooks prepare the meals, the hospital is a favourite one of the French troops.

### A MOTTO FOR HOSPITAL EDITORS.

THE *Gazette* of the 3rd London General Hospital, Wandsworth, opens its second half-year—that is to say, its seventh number—with a new cover design. The artist, however, is the same, Private Ware, R.A.M.C. (T.) The magazine is still rich in illustrations. Private Paul Kirk is again represented, and even Mr. Nevinson, as he now is, has left another Futurist design behind him, which duly appears, although it is reported that, since his return to civil life, his interest in Futurist work has diminished and that he is seeking a new mode of expression. From a journalist's point of view, the plum of the new number is a series of photographs of Queen Amélie, who, we are duly reminded, has declined to gratify the repeated attempts of the Press agencies to secure a photograph of her at her work. Certainly the hospital deserves to have one, the interest of which is enhanced by the accompanying note contributed by Lieut.-Colonel Bruce Porter. It is satisfactory to learn that the sale of the *Gazette* is now some 3,000 copies a month, and the success indicated by this should be an encouragement to other war hospital staffs to make the most of their material. Among the illustrators, and with the numerous artists on its staff it was naturally exceptional in this respect, is one of the Australian patients, Private Vernon Lorimer, who is a writer as well as a lively draughtsman. What should have proved a useful column is that entitled "Some Helpers of the Hospital," and its series of personal notes are carefully done. The motto on the cover, *In arduis fidelis*, is one that seems peculiarly fitted for every editor of a war hospital magazine.

### A SONNET TO A NURSE.

THE popularity of the war magazine is proving infectious, and the latest recruit among the institutions to this form of chronicle is the Norfolk War Hospital. The first issue (April) has the merit of an attempt at variety. Illustrations, caricature, incidents from the trenches, verse, the short story, these together with a descriptive account of the entertainments complete the whole. Among the verses is a sonnet to a nurse, written by a Canadian, in which, addressing the lady, he says: "You who purport to practise healing's art, Have sent me from you with a broken heart!" The phrase "purport to practise" is full of unconscious humour to those who followed with care some of the criticisms of the College of Nursing, which, greeted with much merriment, were lately made at the meeting held in London. The limits of the province of the physician and the nurse are as hard to define in theory as they are simple to observe in practice. It is not often that a sonnet to a nurse is so full as this of professional controversy. In the same magazine in which it appears the classic joke about cascara is made once more in metrical form. We are glad to see articles on the "Birds of Norfolk," and on the nature of the country where General Smuts has been fighting. Such articles gild the pill of the merely funny stories, some of which have worn rather thin. May we also suggest that every illustration should have its title?

### SHOULD HOSPITALS KEEP FOWLS?

WE dealt last week with the criticism of the National Egg Collection for the Wounded emanating from 154 Fleet Street, London, E.C., which *Truth* has made with acumen and force. £38,000 is stated to have been received by this organisation, and no audited or other accounts have yet been published to show clearly how this large sum has been dealt with. Be this as it may, military and other hospitals where wounded are received have created a large demand for eggs for the patients. Where there is ample space—and this there is in the case of commandeered asylum buildings and grounds, to say nothing of many Territorial and other hospitals—the Commandant might well himself institute or sanction the establishment of a poultry section under the management of some of his officers or the matron. Colonel Bruce Porter, at the 3rd London Hospital at Wandsworth, has procured a number of hens, which have been fed upon the scraps that must of necessity remain over in a large institution, however excellently managed. The results are awaited with deep interest. Our experience shows that the equipment and plant required, where ample ground is forthcoming, to establish a poultry section and to promote an ever-increasing supply of eggs is not very large. Under suitable conditions the prospect of success is good. Poultry-keeping, too, might give useful occupation to a number of patients, some of whom are sure to have had experience in the matter.

### A NAMED WARD AT HUDDERSFIELD.

THE Royal Infirmary, Huddersfield, was recently a centre of much interest locally. Mr. Reginald Hirst, lately deceased, had for some years displayed great interest in the institutions of Huddersfield, and was ever willing to help any object which appealed to him, amongst them being the Royal Infirmary, Huddersfield. His widow, Mrs. Hirst, has lately given £3,000 to the infirmary for the naming of a ward in memory of her husband, to be called "The Reginald Hirst Ward." The managers and the board sanctioned the placing of a tablet recording the fact at the entrance of the ward, and this was unveiled, in the absence through illness of Mrs. Hirst, by Miss Sykes, of Fieldhead, who expressed deep sympathy with the widow and high appreciation of the late Mr. Hirst. The ward was then dedicated, the Rev. W. Outram, Vicar of Kirkburton, conducting the special service, which was attended by members of the board, the honorary medical staff, and a number of intimate friends of the Hirst family. Through the liberality of Mrs. Hirst the patients received some extra comforts, and the inmates of the Hirst Ward were supplied with dainties which they greatly appreciated. It is a matter for congratulation that so substantial a sum as £3,000 should have been contributed for the privilege of naming a ward at the Royal Infirmary, Huddersfield.

### NOTIFIABLE DISEASE IN 1915.

A Local Government Board return, showing the total number of cases of notifiable diseases in England and Wales for 1915, has been issued. Scarlet fever heads the list with 127,086 cases (measles and rubeola were not notifiable in 1915); pulmonary tuberculosis stands next with 73,358. Of diphtheria 53,597 cases were notified; and of erysipelas 23,382. Non-pulmonary tuberculosis accounts for 22,864 notifications, but no other disease reaches five figures. Ophthalmic neonatorum was notified 6,806 times, and enteric fever 6,364 times; cerebro-spinal fever attacked 2,586 victims, the largest county contribution being that of London (624). From these figures the curious may reckon that the reduction of fees for notifications from half-a-crown to a shilling each will save the nation about £23,000 a year; for although institutional cases have always stood at a shilling, and therefore no saving is effected upon them, on the other hand measles and roseola are now notifiable and are very prevalent. The great prevalence of tuberculosis and of erysipelas are perhaps the most surprising features of the list, which clearly illustrates the leeway still to be made up before our hygienic and sanitary conditions can be regarded as making plain sailing.

### DOCTORS' FEES AND MOTOR-CARS.

We have previously remarked on the difficulty which the increased tax on motor-cars will cause to medical men and to their patients. In this connection it is tempting to quote part of a letter which has appeared in the Belfast *Newsletter*, written by the Irish medical secretary, B.M.A. After stating that many Irish doctors in rural areas will now have to substitute horses for "horse-power," Mr. Hennessy reminds the public that "the use of motor-cars by doctors . . . was, for obvious reasons, of far more advantage to their patients than to themselves, as even in normal times the cost of motoring far exceeded that of horse-power, without any corresponding increase of fees." The writer adds that, as anticipated, medical practitioners now find that, owing to the certification of sickness benefit under the Insurance Act in Ireland, their mileage has very much increased, "in many cases as much as four or five times." This is serious and very hard on the profession, but it seems that official steps are being taken in Dublin to deal with the matter.

### THE FUTURE OF THE FRESH AIR FUND.

One of the curious effects of the war has been the difficulties which it has placed in the way of the Fresh Air Fund. This depends, or at least has depended, on considerable transit facilities in the process, for which it is annually responsible, of giving a day, or a fortnight, in the country to the children of our great slum cities. Last year all cheap rates had been cancelled, and all spare rolling-stock had been commandeered. Children could not even be taken by train from London to Bushey Park. The ubiquitous tram here and in the provinces came to the rescue, and even barges, tugs, and lorries were also used. The usual total cost of a day in the country is 9d. per child. Last year in several instances the cost of the day for food and fares was over 1s. The human side of the accounts records that 111,255 children were given a day's holiday, and 3,320 a fortnight's holiday, for £7,709 4s. 4d. Thanks to late-coming subscriptions, and, we may note, to the arrival of "several large amounts from abroad," the fund's reserves are still intact. The system of organised treats for poor children was started by Mr. C. Arthur Pearson in 1902. He raised the money, and Sir John Kirk, of the Ragged School Union, gave his invaluable advice as to the most advantageous way of spending it. In 1908 the plan of fortnightly holidays for special cases was started, and we only wish that this side of the work could continue to flourish and compete in extent with the other. There is no nonsense about a fortnight in the country. Even the weather can hardly spoil that. But "a day," though a wonderful experience for many, is not enough. The Fresh Air Fund should steadily aim at Fresh Air for a Fortnight.

### THE CENTENARY OF THE ROYAL WATERLOO HOSPITAL FOR CHILDREN AND WOMEN.

By invitation of the Lord Mayor, president of the hospital, the centenary of this institution is to be celebrated at the Mansion House on Thursday, April 27, after this issue goes to press. The speakers include the Duchess of Albany, Mr. G. W. E. Russell, and Lord Denman. Lady Tree will recite, and Miss Ruth Vincent and Mr. Ben Davies will sing. No collection is contemplated, but Mr. A. C. Benson has published in the daily Press a long and eloquent appeal for money in aid of the Centenary and Special Needs Fund. During the century of its existence the name of this hospital, which was originally founded as a children's infirmary pure and simple, has been changed several times; but it has retained that part of its name which recalls the stirring times through which England had just passed when the hospital was founded. Mr. Benson's appeal, as might be expected of so distinguished and sympathetic a writer, is of a type rather uncommon in institutional begging; but all the more likely to prove effective, one may suspect, for that very reason.

### A STOCKPORT APPOINTMENT.

The Local Government Board has climbed down in connection with the appointment by the Stockport Board of Guardians of Dr. J. Howie Smith as medical officer of the workhouse and Stepping Hill Hospital. When the Guardians, some six or eight weeks ago, elected Dr. Smith as successor to the late Dr. Barker Bale, the Local Government Board refused to sanction the permanent appointment, on the ground that it was not desirable such a post should be filled, except temporarily, during the war. The Guardians, however, stood firm, and retorted that the permanent appointment of Dr. Smith was in the best interests of the institutions and the nation. The result of their strong stand was seen at the board's meeting on Monday, when a letter was read from Whitehall confirming a permanent appointment.

### A CONSULTING STAFF FOR THE LEEDS UNION INFIRMARY.

The Leeds Guardians recently decided to appoint consulting surgeons at the Union Infirmary. At the last meeting of the Board Mr. Thompson was appointed surgeon, Mr. W. Gough gynæcologist, Mr. Whitehead ophthalmic surgeon, Mr. Sharp surgeon for the ear and throat, and Dr. Bowden radiologist. The Leeds Guardians are to be congratulated upon these appointments. They can now boast of a more varied medical staff than any other Board of Guardians in England. We have pointed out for years past the necessity for specialist appointments at the large Poor-Law infirmaries, and it is gratifying to find that Guardians are at length beginning to realise the truth of our contention.

### THE IMPERFECTIONS OF HUMAN NATURE.

The Bermondsey Board of Guardians have been called upon to deal with one of those troubles at their infirmary which, unfortunately, are none too rare nowadays. They had reason to believe that the wardmaids and the laundrymaids were frequently absenting themselves from duty and sending in medical certificates, most of them for trivial ailments. The medical superintendent of the infirmary was asked to examine the maids, with the result that he was of opinion that, with two or three exceptions, they were in sufficiently good physical condition to carry out the work required of them without any undue strain; and he came to the conclusion, also, that many of their recent illnesses were very trivial ailments. The clerk furnished the Board with a return of the maids' absences from duty, from which it appeared that before the Board granted special war bonuses, in thirteen weeks there were 298 days' illnesses, but when the Board gave the bonus but did not pay it when the employees were away sick, the days fell to 113 in fifteen weeks. Then the Board, in a moment of generosity, decided that the women should have the war bonus paid them when they were away ill, as well as their usual full salary, with the result that in the next seventeen weeks they were away 289 days, the total amount paid for salaries during times of temporary illness being £162. The staff is exceptionally well treated in times of sickness, but it was felt that the maids at the infirmary were taking advantage of the kindness of the Guardians, who have now decided to pay no war bonus to those women away ill from the infirmary. At the end of two months a further report is to be submitted to the Board on the absences. All we can say is that the Bermondsey Guardians have displayed an extraordinary lack of understanding of human nature, or else that they let their hearts rule their heads to the detriment of the unfortunate ratepayers.

### A WAR-TIME AMENDMENT OF THE POOR-LAW SUPERANNUATION ACT.

Under the Poor-Law Superannuation Act of 1896 a superannuated officer appointed to a post under the Poor Law loses his pension whilst holding such office. In ordinary times this provision cannot be considered unjust. At present, when many superannuated Poor-Law officers are capable of serving in capacities for which their past experience eminently fits them, and can thus relieve younger members of the Service for active military work, the clause acts as an undesirable deterrent. Clause IV. of the Local Government (Emergency Provisions) Bill now before Parliament has therefore been introduced to remove this obstacle. It provides that "where a person in receipt of a superannuation allowance under the Poor-Law Officers' Superannuation Act, 1896, is appointed or employed as a temporary substitute for any officer or servant by any authority to whom that Act applies the provisions of Section 6 of that Act shall be construed so as not to deprive him of any such allowance whilst he continues to hold such temporary appointment or employment." When this clause becomes law superannuated Poor-Law medical officers will in many cases have greater inducement to return to their old work and thus enable Guardians to release their successors for work with the R.A.M.C. The concession should be of special value as regards the resident Poor-Law medical posts.

### CHILDREN'S INFIRMARY, CLEVELAND STREET, W

The latest addition to the numerous institutions under the control of the Metropolitan Asylums Board is the Children's Infirmary in Cleveland Street, W. This infirmary has recently been taken over from the Holborn Guardians, and will very shortly receive its complement of new patients. The cases to be admitted will be young children up to the age of seven years who still require nursing and older bedridden cases with chronic ailments. Among them will, we are informed, be babies suffering from gastric and intestinal derangements, and cases of chronic heart disease and persistent paralysis. The institution will, in fact, be a children's hospital with a considerable number of nursing staff, and will thus differ from such institutions as St. Anne's Home and the Children's Home, Hanwell, which are intended only for convalescent cases who are up and about, and where the medical work is done by visiting officers. As we have already recorded, this institution has been placed under the control of Dr. C. Price as resident medical superintendent at the salary of £500 per annum with unfurnished house.

### A HANDBOOK OF TUBERCULOSIS SCHEMES.

The National Association for the Prevention of Consumption and Other Forms of Tuberculosis has succeeded by dint of energetic inquiries in gathering together in the form of a handbook authoritative information concerning the present development of tuberculosis schemes throughout the United Kingdom. Complete information of the details of the schemes now in working order has not been easy to compile, as may well be imagined, but the present handbook provides in a brief and tabulated form, firstly, an index to the progress of the campaign against tuberculosis all over the kingdom; and, secondly, a stimulus to responsible authorities to increased effort. Nothing stimulates such a campaign more effectively than the knowledge of what has been undertaken or achieved in other places. It may be hoped that this handbook will be developed and kept up to date; the difficulties of inducing authorities to return accurate information about their schemes are of no light order, and considerable changes may occur in a short time. As far as can be judged, the information in this handbook is as accurate as can be expected. The tables of schemes are divided into those relating to counties and boroughs of the several divisions of the kingdom, and those of the Metropolitan areas. A brief note is also made in each case of the work of care committees. In the Metropolitan areas there are still a few schemes not yet in working order, and in the case of Westminster the information given is already inaccurate owing to the failure of the City Council to come to terms with St. George's and Charing Cross Hospitals. The paucity of open-air schools is a feature which, war or no war, we hope to see improved before the next edition of the handbook is ready. Their need is very great in the big towns.

### THE SUPPLY OF DUTIABLE COMMODITIES TO HOSPITALS.

Arising out of the promise of the Government to allow hospitals—under conditions which have yet to be defined—to use spirit free of duty in the manufacture of medicinal preparations, it is hinted by the *Wine and Spirit Trade Journal* that an effort may be made by hospital authorities to secure a further concession—namely, a rebate of the duty on dutiable commodities generally. There is certainly nothing outrageously unreasonable in the suggestion; indeed, the granting of a rebate of the tax on such articles as tea, sugar, coffee, and cocoa when supplied to hospitals would be a logical sequence of the spirit tax concession which is now pending. The *Wine and Spirit Trade Journal* does not name these articles, but rather suggests that hospital authorities contemplate asking that potable spirits and wines used as "medical comforts" for patients shall be supplied free of duty. The sincerity of our contemporary is not in doubt, but it would be very doubtful wisdom to take counsel with the wine and spirit interests in this matter, and it would certainly be unwise to confuse the question of the tax on medicinal spirits with that of the duty on potable spirits.

### A PLACE OF "SAFETY"!!!

The out-patient department, notwithstanding its vulnerability to criticism, seems destined to remain with us. The Insurance Act failed to kill it. The war is actually giving it a new period of life. We lately quoted Mr. Nevile Chamberlain's remarks on the number of injuries to the eye from which munition workers sought relief in the Birmingham Eye Hospital. Evidence is now to hand that not only the eye, but the entire body of the munition worker is subject to frequent accident—a fact which might be mentioned to those who like to make wild statements about "the slackers hiding in safety" in these munition works! At Sheffield, for instance, the out-patient department of the Royal Infirmary has been under a spell of very heavy work. Accident cases have increased very considerably: actually 21,236 have received treatment. This is attributed to the number of persons, now engaged in munition works, who have never had previous experience of machine-minding and the handling of tools. Whatever the cause, it is clear that a munition works is hardly "a place of safety" for cowards and weaklings. Mr. Punch might distil the humour of this. Mr. George Morrow might draw it.

### THIS WEEK'S DRUG MARKET.

As yet the drug market has not recovered from the influence of the Easter holidays; in normal times the holiday feeling might affect business for several weeks, but under present conditions the market will not remain inactive for many days together. Among the drugs which buyers should carefully watch are the bromides; for some little time the demand has been distinctly quiet, and prices have not been quite so firmly maintained; the general impression, however, is that we are not likely to see substantially lower quotations in the near future. Salicylic acid and salicylate of soda are also drugs which are attracting attention at the moment by reason of rumours to the effect that British producers will shortly be able to offer at prices materially below those which have been ruling for such a long period. While it would, perhaps, be unwise to make purchases long ahead of immediate requirements, it seems doubtful whether the much-talked-of reduction in value is imminent. The fabulous figures asked for cod-liver oil have had the effect of making this market interesting only as a matter of curiosity. Sugar of milk has an upward tendency in price. The high values of citric and tartaric acids are very firmly maintained. The downward tendency in the value of sulphonal is still noticeable.

